# Intravenous Cyclophosphamide in Myalgic Encephalomyelitis/Chronic Fatigue Syndrome. An Open-Label Phase II Study

**DOI:** 10.3389/fmed.2020.00162

**Published:** 2020-04-29

**Authors:** Ingrid G. Rekeland, Alexander Fosså, Asgeir Lande, Irini Ktoridou-Valen, Kari Sørland, Mari Holsen, Karl J. Tronstad, Kristin Risa, Kine Alme, Marte K. Viken, Benedicte A. Lie, Olav Dahl, Olav Mella, Øystein Fluge

**Affiliations:** ^1^Department of Oncology and Medical Physics, Haukeland University Hospital, Bergen, Norway; ^2^Department of Oncology, Norwegian Radium Hospital, Oslo University Hospital, Oslo, Norway; ^3^Department of Medical Genetics, Oslo University Hospital and Faculty of Medicine, University of Oslo, Oslo, Norway; ^4^Clinical Research Unit, Haukeland University Hospital, Bergen, Norway; ^5^Department of Biomedicine, University of Bergen, Bergen, Norway; ^6^Department of Immunology, University of Oslo and Oslo University Hospital-Rikshospitalet, Oslo, Norway

**Keywords:** myalgic encephalomyelitis, chronic fatigue syndrome, ME, CFS, cyclophosphamide, clinical trial, medical treatment, HLA

## Abstract

**Introduction:** Myalgic Encephalomyelitis/Chronic Fatigue Syndrome (ME/CFS) is a disease with high symptom burden, of unknown etiology, with no established treatment. We observed patients with long-standing ME/CFS who got cancer, and who reported improvement of ME/CFS symptoms after chemotherapy including cyclophosphamide, forming the basis for this prospective trial.

**Materials and methods:** This open-label phase II trial included 40 patients with ME/CFS diagnosed by Canadian criteria. Treatment consisted of six intravenous infusions of cyclophosphamide, 600–700 mg/m^2^, given at four-week intervals with follow-up for 18 months, extended to 4 years. Response was defined by self-reported improvements in symptoms by Fatigue score, supported by Short Form 36 (SF-36) scores, physical activity measures and other instruments. Repeated measures of outcome variables were assessed by General linear models. Responses were correlated with specific Human Leukocyte Antigen (HLA) alleles.

**Results:** The overall response rate by Fatigue score was 55.0% (22 of 40 patients). Fatigue score and other outcome variables showed significant improvements compared to baseline. The SF-36 Physical Function score increased from mean 33.0 at baseline to 51.5 at 18 months (all patients), and from mean 35.0 to 69.5 among responders. Mean steps per 24 h increased from mean 3,199 at baseline to 4,347 at 18 months (all patients), and from 3,622 to 5,589 among responders. At extended follow-up to 4 years 68% (15 of 22 responders) were still in remission. Patients positive for HLA-DQB1^*^03:03 and/or HLA-C^*^07:04 (*n* = 12) had significantly higher response rate compared to patients negative for these alleles (*n* = 28), 83 vs. 43%, respectively. Nausea and constipation were common grade 1–2 adverse events. There were one suspected unexpected serious adverse reaction (aggravated POTS) and 11 serious adverse events in eight patients.

**Conclusion:** Intravenous cyclophosphamide treatment was feasible for ME/CFS patients and associated with an acceptable toxicity profile. More than half of the patients responded and with prolonged follow-up, a considerable proportion of patients reported ongoing remission. Without a placebo group, clinical response data must be interpreted with caution. We nevertheless believe a future randomized trial is warranted.

**Clinical Trial Registration:** www.ClinicalTrials.gov, identifier: NCT02444091.

## Introduction

Myalgic Encephalomyelitis/Chronic Fatigue Syndrome (ME/CFS) is a disease of unknown etiology characterized by post-exertional malaise (PEM) (1, 2), sleep disturbances with inadequate restitution (3), fatigue, pain and sensory hypersensitivity, cognitive and several other symptoms. The diagnosis relies on exclusion of other disorders associated with fatigue, and there are no confirmatory diagnostic tests. Using the Canadian consensus criteria (4), an estimated 0.1% of the population suffer from ME/CFS (5), affecting women 3–4 times more often than men. ME/CFS has profound impact on quality of life for patients and their caretakers (6, 7). The socio-economic costs are high, and there is an urgent need for elucidation of the disease mechanisms, for improved diagnostic approaches, and for rational treatment (8).

We hypothesized that ME/CFS could be a variant of an autoimmune disease, with a role for B-cells and possibly autoantibodies. Several observations suggest that immune dysregulation and low-grade inflammation may be involved in the pathogenesis of ME/CFS (9–11). A review (12) summarizes data indicating autoimmunity as a possible etiological factor. Mechanisms may include dysregulations of cytokines (13), alterations in lymphocyte subsets (14) and presence of autoantibodies (15–17). A study with peptide arrays demonstrated an immunosignature based on serum antibodies that separated ME/CFS cases from healthy controls (18). Also, elderly patients with ME/CFS have an increased risk of B-cell lymphomas, especially marginal zone lymphomas known to be associated with autoimmunity or chronic infections (19). Recent research suggests disturbed turnover of complex lipids, fatty acids and amino acids and impaired energy metabolism as possible features of ME/CFS (20–23), possibly linked to low-grade inflammation (24).

There is evidence for a genetic predisposition in ME/CFS (25, 26). The immunologically important Human Leukocyte Antigen (HLA) genes were previously investigated in small ME/CFS cohorts, and certain class II alleles have been found more prevalent among patients (27–29). A recent study of a larger Norwegian cohort of patients and controls, identified two potential HLA risk alleles, namely HLA-C^*^07:04 and HLA-DQB1^*^03:03 (30).

At present, there is no established treatment for ME/CFS. In our oncology unit, we have observed seven patients with long-standing ME/CFS, who reported significant improvement of their ME/CFS symptoms after chemotherapy for either malignant lymphoma or breast cancer. These seven patients all received chemotherapy including the cytotoxic drugs cyclophosphamide or ifosfamide, and one patient also received rituximab. We decided to pursue these observations in separate clinical trials.

Rituximab is a monoclonal antibody that targets CD20 on the surface of B-cells, resulting in reversible B-cell depletion (31). Initial small studies testing rituximab in ME/CFS (32–34) indicated that a subgroup could benefit from B-cell depletion. However, in a recent Norwegian multicenter, randomized, double-blind and placebo-controlled trial, we reported no significant outcome differences between the rituximab and placebo groups (35).

Cyclophosphamide, an alkylating agent widely used in cancer treatment (36), induces immunosuppression and is also used to treat immune-mediated diseases like systemic lupus (SLE), rheumatoid arthritis, vasculitis, and multiple sclerosis (37–40). Based on the assumed immune disturbance in ME/CFS, the observed improvement in ME/CFS symptoms could be due to the immunosuppressive effect of cyclophosphamide (41).

In 2014, we treated four ME/CFS patients with six infusions of cyclophosphamide every 4 weeks. Two of the patients reported substantial improvement of their ME/CFS symptoms, lasting more than 4 years for one of them. In these pilot experiences, there were no infections, neutropenia, thrombocytopenia or unexpected adverse events. We decided to conduct a prospective trial to further investigate feasibility, efficacy and safety of cyclophosphamide treatment in ME/CFS patients.

## Methods

### Trial Design

The CycloME study (EudraCT no. 2014-004029-41, ClinicalTrials.gov NCT02444091) was designed as an open-label phase II trial comprising 40 patients with ME/CFS. The study was approved by the Regional Committees for Medical and Health Research Ethics (2014/1672) and by the National Medicines Agency in Norway. Originally planned for 18 months follow-up, the protocol was amended for prolonged observation of patients up to 4 years after start of treatment. The protocol is available as supporting information (Data Sheet 1).

### Setting and Patient Inclusion

Since 2011 patients with a likely diagnosis of ME/CFS have been referred to the Department of Oncology and Medical Physics, Haukeland University Hospital (HUH), for possible inclusion in clinical trials. Based on available information and proximity to the treating hospital, patients previously included in trials with rituximab and newly referred patients were invited to receive information about the trial. Following signed informed consent, the patients were screened for eligibility.

Inclusion criteria were: a diagnosis of ME/CFS according to the Canadian criteria (4); age 18–66 years; disease duration more than 2 years; and disease severity mild-to-moderate, moderate, moderate-to-severe, or severe. Patients with either mild or very severe disease (completely bedbound and in need of help for all basic activities of daily living) were not included. The exclusion criteria and pre-treatment evaluation are detailed in the trial protocol (Data Sheet 1).

Recruitment lasted from March 2015 until December 2015. All 40 patients were included at the Department of Oncology and Medical Physics, HUH. Seven patients had parts of their treatment and follow-up at the Department of Oncology, Oslo University Hospital (OUH).

Follow-up was originally completed in August 2017, with assessments for prolonged follow-up performed in January 2018 and April 2019.

### Patient Registrations

At baseline, patients recorded severity of a range of common ME/CFS symptoms including PEM, fatigue, cognitive symptoms and pain, using a numerical rating scale of 1–10. During 18 months follow-up, patients were asked to complete a symptom questionnaire every 2 weeks, recording change or no change to the same range of symptoms. The relative scale for symptom change ranged from 0 to 6, in which three denoted no change from baseline; 4, 5, and 6 slight, moderate, and major improvement; and 2, 1, and 0 slight, moderate, and major worsening, respectively. This scale was adapted from the validated Clinical Global Impression Scale, which has been used previously in ME/CFS (42). The primary outcome variable Fatigue score, which has not been validated, was calculated every second week during follow-up as the mean change score for the four fatigue-related items: “Fatigue,” “PEM,” “Need for rest,” and “Daily functioning.” At baseline and every 2 weeks, patients also recorded their percent function level on a scale from 1 to 100%, where 100% denoted a completely healthy state. A set of examples was provided to facilitate this assessment. Samples of all questionnaires are enclosed under Supporting Information (Data Sheet 1). Outcome measures also included the Short Form 36 Health Survey (SF-36) ver. 1.2 in Norwegian translation (43, 44), at baseline, every 3 months during follow-up and at extended follow-up assessments at 24–30 and 38–48 months. Fatigue Severity Scale was recorded at 3-months intervals until 18 months (45, 46). Physical activity level was recorded using an electronic SenseWear armband continuously for 5 to 7 days in a home setting (47, 48), at baseline and repeated in the time intervals 7–9, 11–12, 17–18, 24–30, and 38–48 months after start of treatment.

### Intervention and Follow-Up

Six 30-minute intravenous infusions of cyclophosphamide were administered at 4-week intervals with 600 mg/m^2^ at the first and 700 mg/m^2^ at further cycles. Patients received premedication with ondansetron 8 mg and dexamethasone 4 mg, when necessary enforced by aprepitant 125 mg day 1, and 80 mg days 2 and 3. Patients with hematuria or dysuria in previous cycles were given oral uromitexan (Data Sheet 1). Patients used cold-caps (Elasto-Gel®, Southwest technologies, North Kansas City, USA) during infusions to reduce hair thinning. Each infusion was preceded by routine blood tests, including hematology, and a visit with a physician or study nurse. After the first and second infusions, a nadir blood sample was collected between days 10 and 14 after infusion. If there were no signs of neutropenia or thrombocytopenia after the first two treatments, no further blood tests between treatments were required. Throughout the 18 months follow-up, patients attended consultations with an investigator every 3 months. Adverse events were registered continuously at each treatment visit and at follow-up every 3 months and summarized according to Common Toxicity Criteria for Adverse Events (CTCAE) ver. 4.03. The Viedoc® electronic CRF system (PCG Solutions) was used for data collection and management in the study. There were no interim analyses. The trial was externally monitored by the Department for Research and Development at HUH.

### Outcomes

Response to treatment was defined as Fatigue score ≥4.5 for a minimum of 6 consecutive weeks, occurring at any time point during treatment or within 18 months follow-up. The trial had two primary endpoints based on this definition: (i) overall response rate and (ii) changes in Fatigue score compared to baseline through 18 months follow up. These endpoints were also analyzed separately for the treatment-naïve patients (with no previous rituximab exposure).

Secondary endpoints included: (i) response duration calculated as the sum of response periods each of at least six consecutive weeks with mean Fatigue score ≥4.5; and changes from baseline to specific timepoints of (ii) SF-36 scores for Physical Function subscale (SF-36-PF) and Physical component summary score (SF-36-PCS); (iii) self-reported percent function level; (iv) mean number of steps per 24 h. Adverse events during the 18 months of follow-up from start of treatment were an additional secondary endpoint.

### HLA Typing

High-resolution HLA genotyping was conducted as part of a larger study (30). In short, HLA-A, -B, -C, -DRB1, -DQB1, -DQA1, and -DPB1 alleles were genotyped using NGSgo kits and NGSengine software from GenDX (Utrecht, the Netherlands), and 2 ×150 bp paired-end sequencing on a Miseq instrument (Illumina, San Diego, USA) at the Norwegian Sequencing Centre, Oslo. The association analysis between HLA risk alleles and clinical response was not specified in the protocol, and was performed retrospectively in the data analysis phase. Only the potential HLA risk alleles identified by Lande et al. (30), i.e., HLA-C^*^07:04 and HLA-DQB1^*^03:03, were investigated.

### Statistical Analysis

Descriptive methods were used to characterize the sample, with mean and standard deviation (SD) for normally distributed data, and median with range [min-max, or interquartile range (IQR)] for skewed data. Primary and secondary outcome measures were analyzed by the intention-to-treat principle. Changes from baseline through 18 months follow-up were assessed by General Linear Model for repeated measures (GLM), including time as a predictor. Greenhouse-Geisser corrections were used for all GLM analyses because Mauchly's tests were significant (*p* <0.001), indicating violations of the sphericity assumption. The changes through follow-up, compared to baseline, were assessed by the within-subjects effects for time. Simple contrasts in the time domain were used to assess the changes from baseline to each specific time interval or time point during follow-up, with the effect sizes from the parameter estimates [means and 95% confidence intervals (CI)]. To assess differences between groups GLM repeated measures were performed with *p*-value (Greenhouse-Geisser corrected) from the interaction time-by-group. Groups analyzed were sex, ME/CFS severity, ME/CFS duration, previous rituximab treatment, infection prior to debut of ME/CFS symptoms, and specific HLA alleles. The distribution of sex, ME/CFS severity and the proportion of responders among carriers and non-carriers of the two aforementioned HLA-alleles, were compared using Odds Ratio (OR) and Fisher's exact tests.

All tests were two-sided with a significance level of 0.05. Missing data were replaced using the last value carried forward (LVCF) method. All analyses were performed using IBM SPSS Statistics ver.25 (IBM Corp., Armonk, USA), and Graphpad Prism ver.8 (GraphPad Software, La Jolla, USA).

### Role of the Funding Sources

The research group for ME/CFS at Department of Oncology and Medical Physics (HUH) has received funding from the Kavli Trust and the Norwegian Ministry of Health and Care Services. The HLA sequencing has received funding from the Kavli Trust and Norwegian Research Council. The funders had no role in trial design, data collection, analysis, decision to publish, or preparation of the manuscript.

## Results

### Study Population

**Figure 1:**
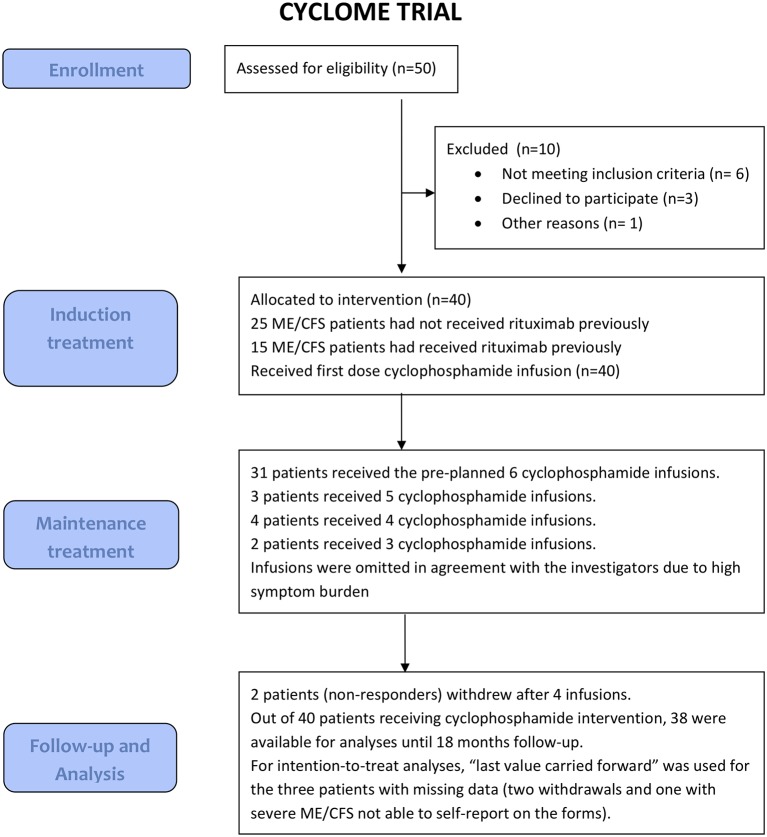
Flow diagram of enrollment, treatment and follow-up in the CycloME study.

The flow chart for patient screening, inclusion, treatment and follow-up is shown in Figure 1. Among available referrals with adequate medical information, we randomly selected 50 patients for eligibility screening. Ten patients were excluded due to violation of eligibility criteria, or declined to participate. We included 25 rituximab-naïve patients and 15 patients with previous rituximab intervention.

Table 1 shows baseline characteristics for all included patients (*n* = 40), the rituximab-naïve patients (*n* = 25), and patients with (*n* = 22) or without (*n* = 18) a response to cyclophosphamide according to the definition of the primary endpoint of the study.

**Table 1:** Baseline characteristics of the study population are shown for the intention-to-treat population, for rituximab-naïve patients and for patients with or without clinical response.

**Characteristic**	**All patients (*n* = 40)**	**Rituximab-naïve^**a**^ (*n* = 25)**	**Responders^**b**^ (*n* = 22)**	**Non-responders^**c**^ (*n* = 18)**
Female, *n* (%)	31 (77.5)	18 (72.0)	18 (81.8)	13 (72.2)
Male, *n* (%)	9 (22.5)	7 (28.0)	4 (18.2)	5 (27.8)
Age, female pts, mean (min–max)	43.0 (25.0–61.1)	41.5 (26.6–54.6)	41.8 (25.0–60.3)	44.6 (26.6–61.1)
Age, male pts, mean (min–max)	37.6 (21.5–53.3)	35.1 (21.5–50.8)	39.5 (21.5–53.3)	36.0 (23.4–50.8)
BMI female pts^d^, mean (min–max)	24.5 (17.1–33.1)	24.6 (17.1–33.1)	24.1 (17.1–32.7)	24.9 (19.0–33.1)
BMI male pts^d^, mean (min–max)	24.5 (17.4–30.6)	23.4 (17.4–29.2)	25.9 (17.4–30.6)	23.4 (21.1–26.9)
Rituximab-naïve^a^, *n* (%)	25 (62.5)	25 (100.0)	14 (63.6)	12 (66.7)
Previous rituximab treatment^e^, *n* (%)	15 (37.5)	0	9 (40.9)	6 (33.3)
**ME/CFS disease duration**				
2–5 years, *n* (%)	7 (17.5)	7 (28.0)	5 (22.7)	2 (11.1)
5–10 years, *n* (%)	13 (32.5)	7 (28.0)	5 (22.7)	8 (44.4)
10–15 years, *n* (%)	9 (22.5)	4 (16.0)	6 (27.3)	3 (16.7)
>15 years	11 (27.5)	7 (28.0)	6 (27.3)	5 (27.8)
**ME/CFS disease severity**				
Mild/Moderate, *n* (%)	14 (35.0)	10 (40.0)	9 (40.9)	5 (27.8)
Moderate, *n* (%)	13 (32.5)	7 (28.0)	9 (40.9)	4 (22.2)
Moderate/severe, *n* (%)	7 (17.5)	5 (20.0)	4 (18.2)	3 (16.7)
Severe^f^, *n* (%)	6 (15.0)	3 (12.0)	0	6 (33.3)
Infection prior to ME/CFS^g^, *n* (%)	26 (65.0)	17 (68.0)	15 (68.2)	11 (61.1)
SF36 Physical Function^h^, mean (min–max)	33.0 (0–65)	34.0 (0–65)	35.0 (10–65)	30.6 (0–65)
SF36 Physical component summary scorex^i^, mean (min–max)	23.3 (13.5–41.6)	24.5 (14.6–41.6)	23.1 (13.5–41.6)	23.5 (14.6–31.0)
Steps, mean per 24 h, mean (min–max)	3,199 (568–9,637)	3,282 (568–9,637)	3,622 (1,083–8,178)	2,681 (568–9,637)
Total function level^j^, mean (min–max)	16.9 (5–40)	17.0 (5–30)	19.3 (10–40)	14.1 (5–25)
HLA-DQB1*03:03 pos, *n* (%)^k^	10 (25.0)	6 (24.0)	9 (40.9)	1 (5.6)
HLA-C *07:04 pos, *n* (%)	4 (10.0)	2 (8.0)	3 (13.6)	1 (5.6)
HLA-DQB1*03:03 and/or HLA-C*07:04 pos, *n* (%)	12 (30.0)	6 (24.0)	10 (45.5)	2 (11.1)

a *Patients with no previous rituximab intervention*.

b Clinically significant responders, including 18 patients with long response duration (≥30 weeks), three with moderate response duration (14–28 weeks) and one with marginal response duration (6–12 weeks).

c *Patients with no clinically significant response*.

d *Body Mass Index (kg/m^2^)*.

e *Patients treated with rituximab in previous trial (KTS-2-2010) n = 14, or outside a clinical trial (n = 1)*.

f *Two of six patients with severe ME/CFS withdrew from the study after four infusions*.

g *Self-reported infection prior to onset of ME/CFS disease*.

h *Short Form 36 (SF-36) physical function subscale (scale 0–100)*.

i *SF-36 Physical Health Summary Score, norm-based with population mean 50*.

j *Baseline self-reported function level (scale 0–100%)*.

k *HLA-types determined as part of a larger study (30)*.

Medical history and concomitant diseases at baseline, and concomitant medication during study follow-up, are summarized in Supplementary Tables 1, 3. Supplementary Table 2 shows previous treatment by trial participants. Some kind of cognitive therapy had been tried by 52.5%, graded exercise or other physical therapy by 45.0%, adaptive pacing by 37.5%, vitamin B12 injections by 40.0%, and low dose naltrexone by 37.5%. None of the patients received any alternative intervention aimed at ME/CFS during the trial.

Thirty-one patients received all preplanned six infusions, three patients received five infusions, four received four infusions, and two received three infusions (Figure 1). The reasons for omitting infusions were either withdrawal of consent (two cases after cycle 4), or high symptom burden (seven cases). All the decisions to omit infusions were in agreement with the trial investigators. Thus, nine patients (22.5%) deviated from the planned treatment protocol.

### Missing Data

For the 18 months study period, there were missing data for the two patients who withdrew from study after ~5 months (both non-responders at the time of withdrawal), and for one non-responding patient with severe ME/CFS who failed to complete self-reported forms from 4 months onwards. Except for these three patients, there were eight missing data items out of 1,560 raw data for the variable Fatigue score. SenseWear activity armband data were complete at baseline, and had missing data from the two withdrawals during follow-up.

### Primary Outcome

The overall response rate, i.e., proportion of patients with Fatigue score ≥4.5 for at least six consecutive weeks, was 22 out of 40 patients (55.0%, 95% CI 39.8–69.3%). Among the rituximab-naïve patients, 14 out of 25 patients achieved a clinical response (56.0%, 95% CI 36.9–73.4%).

**Figure 2:**
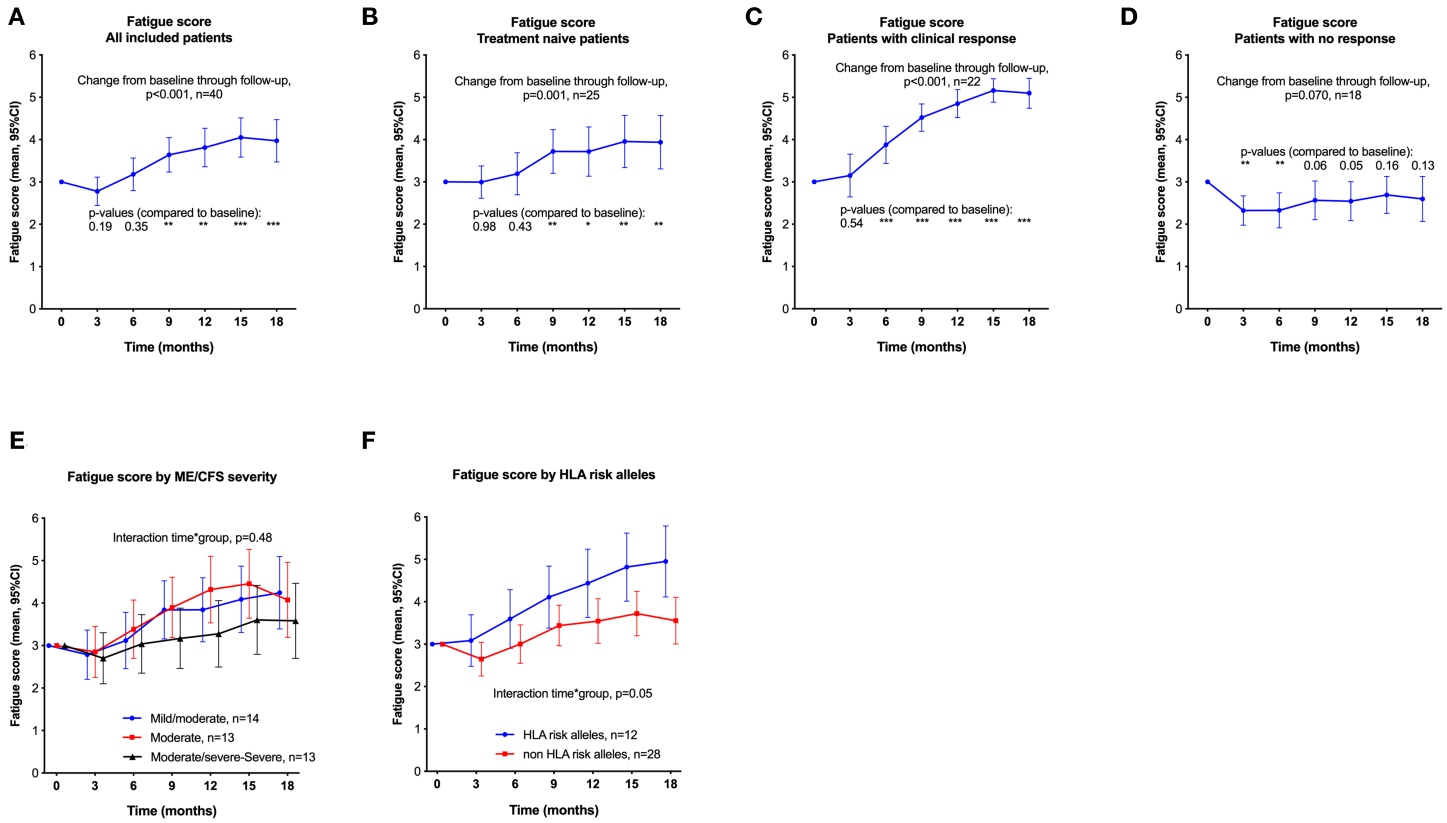
Fatigue score (primary end point), means with 95% CI at time points through 18 months follow-up, from self-reported symptom scores every second week. The scale is 0–6, where 3 indicates no change from baseline and higher scores indicate less fatigue. **(A)**: All included patients (*n* = 40). **(B)**: Treatment-naïve patients (not previously exposed to rituximab, *n* = 25). **(C)**: Responders during follow-up (*n* = 22). **(D)** Non-responders during follow-up (*n* = 18). *P*-values from General Linear Model repeated measures assessing changes in Fatigue score from baseline. **(E,F)** show mean Fatigue score (with 95% CI) through 18 months' follow-up, subgrouped by ME/CFS disease severity **(E)**, and presence/absence of HLA risk alleles **(F)**. *P*-values from General Linear Model for interaction time-by-group, assessing difference between subgroups in repeated measures of Fatigue score over time compared to baseline. *P*-values: * <0.05; ** <0.01; *** <0.001. CI, confidence intervals.

Changes in Fatigue score during 18 months follow-up, with comparisons of mean Fatigue score at each 3-month interval to baseline are shown in Figure 2, for all patients (Figure 2A), rituximab-naïve patients (Figure 2B), patients with a response (Figure 2C), and no response during follow-up (Figure 2D). Repeated measures of Fatigue score showed significant increases from baseline, with similar improvements among the rituximab-naïve patients as observed in all patients. The Fatigue score increased significantly from baseline to 9 months after start of treatment and further through 18 months follow-up. Among the 18 patients with no response, the Fatigue score decreased significantly from baseline to 3 and 6 months, and thereafter returned to near baseline level. Figure 2 also shows the courses of mean Fatigue score through 18 months' follow-up, subgrouped by ME/CFS disease severity (Figure 2E), and by presence/absence of HLA risk alleles (Figure 2F) in which patients with HLA-DQB1^*^03:03 and/or HLA-C^*^07:04 reported higher improvements of Fatigue score through follow-up than those negative for these alleles (*p* = 0.05).

### Secondary Outcomes

**Figure 3:**
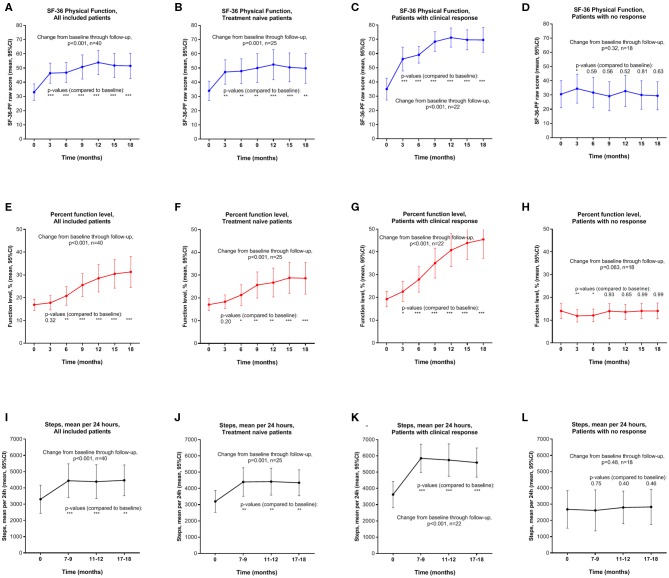
SF-36 Physical Function (SF-36-PF) **(A–D)**, percent function level **(E–H)**, and mean steps per 24 h **(I–L)**, means with 95% CI, at time points through 18 months follow-up. Outcome data for all included patients (*n* = 40) **(A,E,I)**; Treatment-naïve (not previously exposed to rituximab, *n* = 25) **(B,F,J)**; Responders during follow-up (*n* = 22) **(C,G,K)**; Non-responders during follow-up (*n* = 18) **(D,H,L)**. *P*-values from General Linear Model repeated measures assessing changes in outcome variable from baseline. *P*-values: * <0.05; ** <0.01; *** <0.001. SF-36 Physical Function with scale 0–100, higher number indicates better function. CI, confidence intervals; SF-36, Short Form 36.

Changes of SF-36-PF and percent function level through each 3-month interval, and mean steps per 24 h (at baseline, 7–9, 11–12, and 17–18 months), are shown in Figures 3A–L. Outcomes are shown for all patients and for the rituximab-naïve group, as well as for patients with and without response according to the study criteria. There were significant improvements of all outcome variables from baseline through 18 months follow-up among all 40 patients, with mean SF-36-PF increasing from 33.0 at baseline to a maximum 51.5 at 18 months follow-up (*p* <0.001). Among 25 rituximab-naïve patients, mean SF-36-PF increased from 34.0 at baseline to 49.8 at 18 months (*p* = 0.001). Among 22 responders, mean SF-36-PF increased from 35.0 at baseline to 69.5 at 18 months (*p* <0.001). For 18 non-responders there was only a slight increase of SF-36-PF from 30.6 at baseline to a maximum of 34.4 at 3 months, and with no significant changes through the remaining study follow-up. Similar patterns of significant changes were seen through follow-up, as compared to baseline, for percent function level and for mean steps per 24 h, and also for SF-36-PCS (not shown).

**Figure 4:**
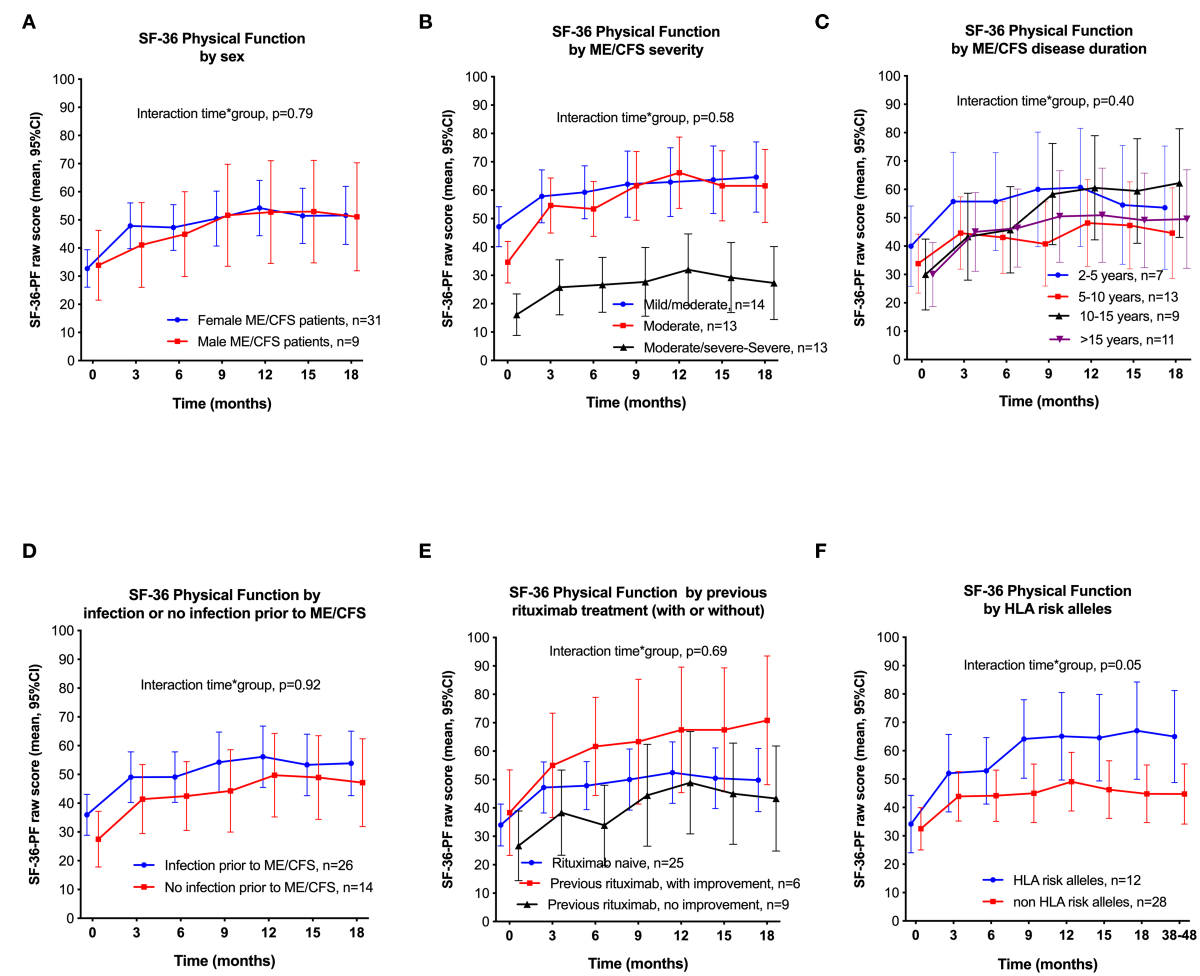
SF-36 Physical Function (SF-36-PF), means with 95% CI through 18 months follow-up, by subgroups. *P*-values from General Linear Model for interaction time-by-group, assessing differences between the subgroups in repeated measures of SF-36-PF over time, compared to baseline. **(A)**: By men vs. women; **(B)** By ME/CFS severity; **(C)**: By ME/CFS disease duration; **(D)** With or without self-reported infection prior to ME/CFS; **(E)** With or without previous rituximab treatment; **(F)**: With or without HLA risk alleles (HLA DQB1*03:03 and/or HLA C*07:04). SF-36, Short Form 36; CI, confidence intervals; HLA, Human Leukocyte Antigen.

Figure 4 shows the courses of SF-36-PF by subgroups. There were no significant interactions time-by-group for sex, severity, disease duration, infection prior to ME/CFS, or previous treatment with rituximab, i.e., the changes in SF-36-PF over time were similar in all subgroups, except for HLA risk allele defined subgroups (see below). The reason for showing SF-36-PF in these plots was to enable comparison of data to other reported studies, in which SF-36-PF has often been used. There was no significant overall interaction between time and ME/CFS severity (*p* = 0.51), although the small group (*n* = 6) with severe disease had no clinically relevant increase in SF-36-PF, from 8.3 at baseline to a maximum of 11.7 at 12 months follow-up. The severe ME/CFS group included two patients with missing data (one withdrawal and one who failed to complete registration). However, seven patients with moderate-to-severe disease had similar improvements of the outcome measures as patients with either moderate or mild-to-moderate disease. Supplementary Figure 1 shows the courses during follow-up, for the SF-36 subscales Vitality, Social Function, and Bodily Pain (Supplementary Figures 1A–F), and also the Fatigue Severity Scale (Supplementary Figures 1G,H), all showing that the responders report improvement during follow-up which we interpret to be of clinical significance.

Out of nine patients included in the trial who had received previous rituximab treatment without reporting improvement of ME/CFS symptoms, four achieved a clinical response after cyclophosphamide intervention. Patients with HLA alleles HLA-DQB1^*^03:03 and/or HLA-C^*^07:04 reported higher improvements of SF-36-PF through follow-up than those negative for these alleles (*p* = 0.05) (Figure 4F).

### Clinical Response Durations

Among the 22 patients with response, the total duration or response was median 44 weeks (range 6–70 weeks) within 18 months follow-up. The median ratio of clinical response duration to follow-up was 0.56 (range 0.08–0.90). Response duration was ≥ 30 weeks in 18 patients, 14–28 weeks in three patients, and 6–12 weeks in one patient.

The median time to first response was 22 weeks (range 2–42 weeks). There were no significant differences in time to first response by sex, disease severity, disease duration, infection prior to ME/CFS, or by previous rituximab treatment (data not shown).

Out of 22 responders, 17 patients (77.3%) reported a sustained response with Fatigue score of least 4.5 at the end of 18 months follow-up. Among all 40 included patients, 21 (52.5%) reported a Fatigue score of at least 4.0 (slight improvement) at end of follow-up.

### Prolonged Follow-Up

**Figure 5:**
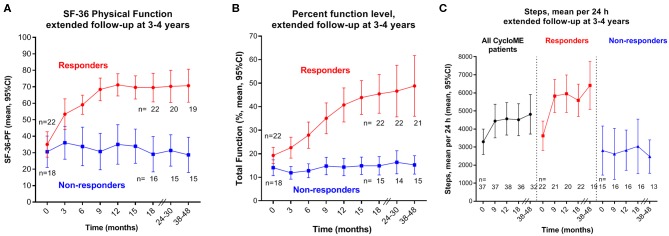
Outcome variables at extended follow-up, by response status. Means with 95% CI at different time points including extended follow-up at 24–30 and at 38–48 months. **(A)**: SF-36 Physical Function (SF-36-PF); **(B)** percent function level; **(C)**: mean steps per 24 h. Numbers of patients at the different time points through follow-up are shown below the graphs. SF-36, Short Form 36; CI, confidence intervals.

Following two approved protocol amendments, patients had additional visits or telephone interviews with recordings of SF-36 and percent function level and SenseWear physical activity measurements at 24–30 and 38–48 months follow-up. Due to the risk of recall bias, Fatigue score compared to baseline was not recorded at these late visits. Instead, patients were asked to self-assess whether their symptoms had relapsed, remained unchanged or had improved further since the end of trial (18 months). The changes of SF-36-PF, percent function level and mean steps, from baseline until extended follow-up at 38–48 months, by response status, are shown in Figures 5A–C.

At the 38–48 months visit, 36 out of 38 patients still in the study completed the interview including assessment of their percent function level, 35 recorded SF-36 forms and 32 completed SenseWear activity measurements. Out of 22 responders, 20 completed the interview; 15 were still in remission, while five reported a complete or partial relapse. For 20 responders with available SF-36 recordings at 38–48 months, the mean SF-36-PF was 70.8 (range 25–100) compared to mean 69.5 at 18 months. SenseWear activity registration was available for 19 out of 22 responders at 38–48 months with mean 6,415 steps per 24 h (SD 2,764), compared to mean 5,589 (SD 2,017) at 18 months (Figure 5C). Six patients with missing SenseWear data at 38–48 months included two responders in ongoing remission, one in relapse and three non-responders.

At baseline, only two of the responders had part-time work participation. During follow-up, at least nine out of 22 responders returned to either part-time of full-time work or studies.

### HLA Data

**Figure 6:**
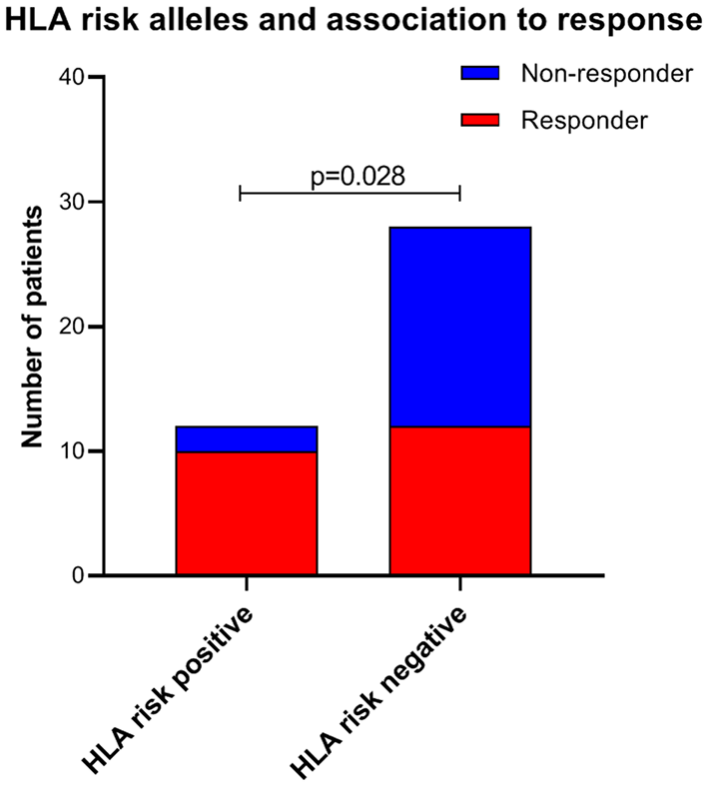
Frequency of HLA risk alleles (HLA DQB1*03:03 and/or HLA C*07:04) in responders and non-responders during follow-up. *P*-value from Fischer's exact test. HLA, Human Leukocyte Antigen.

Twelve of the 40 patients (30.0%) carried either of the two specific HLA risk alleles. Ten of the 12 patients (83.3%) positive for HLA alleles DQB1^*^03:03 and/or C^*^07:04 had a response, compared to 12 out of 28 patients (42.9%) negative for these HLA alleles (OR = 6.67; *p* = 0.028; Figure 6). The allele HLA-C^*^07:04 was present in four out of 40 patients (10.0%), and three (75.0%) of these were responders. HLA-DQB1^*^03:03 was detected in 10 out of 40 patients (25.0%), and 9 out of 10 (90.0%) were responders, compared to 13 out of 30 HLA-DQB1^*^03:03 negative patients (43.3%); OR = 11.8, *p* = 0.013.

Among 12 patients with the HLA alleles DQB1^*^03:03 and/or C^*^07:04, 7 (58.3%) had mild-to-moderate, 3 (25%) had moderate, and 2 (16.7%) had moderate-to-severe ME/CFS. Contrary, among 28 patients without these HLA alleles, 7 (25.0%) had mild-to-moderate, 10 (35.7%) had moderate, and 11 (39.3%) had moderate-to-severe ME/CFS (*p* = 0.05). Eleven out of 12 patients (91.7%) with HLA alleles DQB1^*^03:03 and/or C^*^07:04 were female, compared to 20 out of 28 patients (71.4%) without these alleles (*p* = 0.23).

### Adverse Events

Adverse events (AE) for the complete period of 18 months follow-up are shown in Table 2. Thirty-three patients (82.5%) reported AEs of CTCAE grade ≥ 2, of which gastrointestinal events such as nausea and constipation were the most common. Out of 16 grade 3–4 events in 11 patients, 11 resulted in hospitalization and were reported as serious adverse events (SAE, Supplementary Table 4). There was one suspected unexpected serious adverse reaction (SUSAR), in a female patient with moderate-to-severe ME/CFS who was a non-responder in the study. She experienced gradual worsening of postural orthostatic tachycardia syndrome (POTS) after cycle 4, resulting in hospital admission for 2 weeks. She had experienced periods of similar POTS aggravations regularly since she became ill with ME/CFS 18 years before study inclusion. Her POTS symptoms gradually returned to baseline level, but study treatment was discontinued. With routine blood sampling before each cycle and after cycle 1 and 2, there was no sign of hematological toxicity. Two women both aged ≥41years at inclusion, experienced menopause after start of treatment, two others reported irregular menstrual bleeding that persisted to end of follow-up. One patient without a clinical response suffered a sudden death of unknown cause 4 years after inclusion in the study, i.e., 42 months after the last infusion, with no probable relation to the intervention.

**Table 2:** Patients with adverse events of CTCAE grade 1–4 during 18 months follow-up.

	**≥1**	**≥2**	3–4^*^	**Related to study treatment^**†**^**
Patients with ≥ 1 adverse event	39 (97.5%)	33 (82.5%)	11 (27.5%)	29 (72.5%)
Nausea	36 (90%)	15 (37.5%)	0	36 (90%)
Constipation	22 (55%)	9 (22.5%)	1 (2.5%)	19 (47.5%)
Diarrhea	7 (17.5%)	1 (2.5%)	0	6 (15%)
Stomach pain	9 (22.5%)	2 (5%)	1 (2.5%)	7 (17.5%)
Infections	24 (60%)	15 (37.5%)	3 (7.5%)	13 (32.5%)
Irregular menstrual bleeding	7 (17.5%)	3 (7.5%)	0	7 (17.5%)
Premature menopause	2 (5%)	1 (2.5%)	0	2 (5%)
Haematuria	6 (15%)	1 (2.5%)	0	6 (15%)
Urinary bladder symptoms^**^	5 (12.5%)	3 (7.5%)	0	5 (12.5%)
Hair loss	4 (10%)	0	0	4 (10%)
Rash or urticaria	6 (15%)	4 (10%)	1 (2.5%)	5 (12.5%)
Headache	12 (30%)	3 (7.5%)	1 (2.5%)	9 (22.5%)
Dizziness	6 (15%)	3 (7.5%)	0	5 (12.5%)
Edema of face or limbs	6 (15%)	0	0	5 (12.5%)
Palpitations or tachycardia	4 (10%)	2 (5%)	1 (2.5%)	2 (5%)

* *11 grade 3–4 events for 8 patients were reported as SAE. See Supplementary Table 4 for details*.

† *Possible, probable or very likely relation to study treatment*.

** *Bladder/urinary tract pain or increased urinary frequency*.

## Discussion

The present open-label phase II study with cyclophosphamide infusions was well conducted with little missing data. More than half of the patients had clinical response according to the predefined criteria, many with long-lasting improvement of symptoms. At extended follow-up 3–4 years after inclusion 68% of responders were still in remission.

In general, the toxicity to cyclophosphamide infusions in ME/CFS patients was moderate, and there were few serious adverse events and no registered hematological toxicity. The most common side effects were nausea and general malaise lasting for 1–2 weeks after each infusion. ME/CFS patients reported more nausea and discomfort after cyclophosphamide than cancer patients typically do at similar doses, in line with the generally low stress tolerance and sensitivity to drugs reported by many patients. We reinforced the anti-emetic regimen with aprepitant during the study in efforts to reduce the nausea experienced by the patients during the first days after infusion. Fertility concerns are an important toxicity issue with chemotherapy. Cyclophosphamide is an alkylating agent associated with ovarian failure and the risk increases with higher cumulative doses and with increasing age (49, 50). One study with intravenous infusions, applying similar cumulative doses (mean 9.1 gram) as in the present study, and mean age 31 years, reported ovarian failure in 13%, and transient amenorrhea in 20% of the patients (51). In our present trial, two women aged 41 and 46 years at inclusion experienced premature menopause, and two others reported irregular menstruation probably induced by the treatment at end of follow-up. In contrast to spontaneous premature menopause, chemotherapy associated ovarian dysfunction can resume over time (years) in some patients, even after a prolonged period of amenorrhea and elevated gonadotropin levels (52).

Since the 6-month initial treatment period with repeated cyclophosphamide infusions in some patients led to increased symptom burden and side effects, the extent and duration of improvement in ME/CFS symptoms are important aspects to justify the intervention. We therefore extended the follow-up period, and collected additional clinical data from participants, at 2–3 and 3–4 years after inclusion. The response durations were sustained for most of the responders. Out of 22 responders 82% were still in remission at 2–3 years and 68% at 3–4 years extended follow-up. Seven even reported further improvement compared to their status at 18 months follow-up. Also of note, three of the patients who registered relapse at 3–4 years still reported a 2-fold increase of their percent function levels as compared to baseline. Thus, responders' self-reported percent function levels, SF-36 Physical Function with increase from mean 35 at baseline to mean >70 at 12 months, and measured levels of physical activity (steps per 24 h), reflect clinically meaningful improvements of their abilities and activities of daily life. For comparison, the mean SF-36 Physical Function in the general population is 84.2 (95% CI 71.9–96.5) (53).

Compared to the randomized RituxME trial assessing rituximab vs. placebo in ME/CFS patients (35), the patterns of improvement among patients in the present CycloME trial seemed to be more homogeneous. In CycloME the clinical responses occurred earlier than in the RituxME trial; at median 22 weeks compared to 41 weeks. In the CycloME study the response rates were comparable between men and women, as opposed to higher response in women in the RituxME trial. The response rates were higher among patients with moderate or moderate-to-severe disease, compared to the 4 patients with severe ME/CFS who completed the intervention. In an ongoing addition to the trial (part B), feasibility and response rate are investigated in a small number of additional patients with severe ME/CFS, to gain experience and to decide whether severe patients may be included in a possible future randomized trial assessing cyclophosphamide intervention.

The response rates were similar among patients who were rituximab-naïve and patients who had participated in previous trials with rituximab intervention (32, 33). Also, four out of nine patients with no improvement after previous rituximab intervention experienced clinical benefit after cyclophosphamide in the present study.

Interestingly, the presence of either of the two HLA risk alleles, previously shown to be associated with ME/CFS (HLA-DQB1^*^03:03 and HLA-C^*^07:04) (30), was predictive for response to cyclophosphamide. In contrast there was no association between presence of these HLA alleles and clinical improvement among patients included in the RituxME trial (35) (data not shown).

The carrier frequency of any of these HLA risk alleles was 30% among ME/CFS patients in this trial, which is higher than the 19.1% reported in the recent study of 426 Norwegian ME/CFS patients (30). Western Norway is well represented in this large cohort, and the frequency of DQB1^*^03:03 and C^*^07:04 from Western Norway sources did not differ from the national frequency (data not shown). Therefore, geographical bias is not a probable explanation.

The association between cyclophosphamide response and the HLA risk alleles could be due to a true treatment effect in individuals carrying these alleles. There are several reports of associations between specific HLA alleles/haplotypes and responses to immune modulatory treatments (54–57), but to our knowledge this has not been demonstrated specifically for cyclophosphamide. Another possibility is that carriers of these HLA risk alleles constitute a subgroup among ME/CFS patients with an immune-driven pathomechanism generally responding better to immune modulating treatment. Finally, the observed association between the HLA risk alleles and response to cyclophosphamide could be coincidental, but warrants further investigation in a possible future randomized trial.

There are no biomarkers for ME/CFS or disease activity, and assessments of symptom changes consequently have to rely largely on self-recorded subjective variables. To increase the validity of the measurements, we used several different variables to measure symptom changes. These variables generally showed the same patterns of improvement and worsening of ME/CFS symptoms during the follow-up period. Self-reported improvements in Fatigue score, percent function level and SF-36 Physical Function scores correlated well, and with increased levels of physical activity. “Steps per 24 hours” is an objective measure, but not a perfect way to validate symptom improvement because individual patients will use their improved energy for different purposes. Some will walk, while some will prefer to read or increase the time for social activity.

The initial patient observations in our cancer clinic, of patients with long-standing ME/CFS who developed cancer, and who reported relief of ME/CFS symptoms after cancer treatment, included seven cases treated with cyclophosphamide (or ifosfamide), and in one case the combination of cyclophosphamide and rituximab. Our hypothesis was that ME/CFS in a subgroup of patients could be caused by an immunological dysfunction, possibly with a variant of an autoimmune pathomechanism. In the present study, the frequency of self-reported infection prior to ME/CFS debut (65%) was in line with other reports (58). Also, there was a high occurrence of autoimmunity among first-degree relatives (55.0%). Both observations may support an immunological basis for the disease. Initial phase II studies with rituximab (32, 33) suggested that a subgroup of patients could benefit from B-cell depletion therapy. Conversely, in the double-blind, placebo-controlled, multicenter, phase III RituxME trial there were no significant differences between the rituximab and placebo groups for any of the primary or secondary outcome measures (35). Taking the RituxME results into account, we have to interpret the data from the present open-label CycloME trial with caution. Patient selection, placebo mechanisms, patient's expectations in clinical trials, and natural variation of symptoms over time may be operative (59, 60). Until a randomized trial has been performed, there is not sufficient evidence for a beneficial effect of cyclophosphamide in ME/CFS patients.

Other study limitations are self-referral, use of self-reported primary outcome measures with possible recall bias, and the inclusion of patients who had participated in previous studies with rituximab intervention. Although inclusion relied on strict diagnostic criteria, the unknown etiology of ME/CFS and lack of specific biomarkers could introduce unintended heterogeneity of the patient sample.

When comparing the response data from the CycloME and RituxME studies, it is important to consider the completely different modes of action of the two drugs. Rituximab is a monoclonal antibody which selectively depletes B-cells expressing the CD20 protein on their surface, while cyclophosphamide has broader immunosuppressive effects on several subsets of lymphocytes. The main mechanism of cyclophosphamide is the ability to covalently bind an alkyl group, affecting mainly the DNA (61). This interaction is irreversible and leads to inhibition of DNA replication and apoptosis, producing cell death amongst resting and dividing white blood cells and leading to impaired humoral and cellular immune responses (62). Rapidly proliferating cells are most sensitive to cyclophosphamide (41). This feature is utilized in cancer therapy, but also to influence activated immune cells that are present in different immune-mediated diseases (37). The effects and side-effects of cyclophosphamide are highly dose dependent. High doses can be used for the complete eradication of hematopoietic cells, but lower doses are relatively selective for T-cells, especially T-regulatory cells (T-regs). Cyclophosphamide affects T-regs, which have a generally higher proliferation rate than other T-cell subsets such as the T-helper (Th) cells, but also affects B-cells and other cells of the immune system (41). T-regs have an important role in down-regulating the effects of Th cells, and help prevent autoimmune diseases by maintaining self-tolerance (63). A higher frequency of T-regs in ME/CFS patients compared to healthy controls has been reported in some studies (64–66). The T-reg markers are also general T-cell activation markers (63). Thus, cyclophosphamide may interfere with the balance between immune cell subsets and possibly counteract a disease-facilitating environment.

Although the double blinded RituxME trial showed no significant differences between the rituximab and placebo groups for the outcome measures (35), there may still be a subgroup of ME/CFS patients that have an autoantibody-mediated disease where only few patients have autoantibody-production from early CD20-positive plasmablasts that can be targeted by rituximab. Other patients may still have autoantibody production, but from long-lived CD20-negative plasma cells. This mechanism is active in several rituximab refractory autoimmune diseases and could be compatible both with the total experience from our rituximab trials, and with the data from the present cyclophosphamide trial. Cytotoxic chemotherapy, such as cyclophosphamide, may inhibit B-cell activation and proliferation to new antibody-secreting cells, thus reducing the short-lived plasma cell compartment and recruitment of mature plasma cells (67).

If an autoantibody-mediated mechanism is operative in a subgroup of ME/CFS patients, the nature of possible endogenous targets for pathogenic immunoglobulins is still elusive. Increased serum levels of autoantibodies against several G-protein coupled receptors have been shown in ME/CFS (16). Clinical symptoms suggest inadequate regulation of autonomic functions and blood flow, also demonstrated in a recent study of reduced cerebral blood flow during head-up tilt test with orthostatic stress using Doppler flow imaging of carotid and vertebral arteries (68). Recent observations of patients with unexplained exertional intolerance and dyspnea demonstrated a subgroup with low ventricular filling pressure (preload failure) in upright position during cardiopulmonary exercise tests, related to reduced venous pressure (69, 70). Also, in patients with unexplained exertional intolerance, a subgroup had impaired systemic oxygen extraction, which may be associated with microcirculatory dysregulation or mitochondrial dysfunction (71). One might speculate on the possibility of an autoimmune process indirectly of directly affecting blood vessels, or against small nerve fibers including autonomic nerves regulating blood vessel function. Small fiber neuropathy (SFN) is associated with fatigue, postural orthostatic tachycardia syndrome (POTS), gastrointestinal disturbances and abnormal sweating (72). SFN has been demonstrated in 49% of fibromyalgia patients (73), and in up to 43% of patients with preload failure, many of whom had symptoms suggestive of ME/CFS (70). This could be associated with inadequate autoregulation of blood flow according to the demands of tissues, with local hypoxia and lactate accumulation on limited exertion, and with metabolic adjustments which could be secondary and compensatory in efforts to restore cellular energy balance (20, 21, 23, 74, 75). Microvasculopathy may also be reflected in arterial endothelial dysfunction which has been demonstrated in ME/CFS (76), and also investigated in substudies to the CycloME and RituxME trials (manuscripts in preparation).

The growing evidence for immune disturbances in ME/CFS, experience with cyclophosphamide in other autoimmune diseases, with broad immunosuppressive effects on several lymphocyte subsets including B-cells and T-regs, and the herein reported association between HLA risk alleles and clinical response to cyclophosphamide intervention, support that the observed relief of ME/CFS symptoms could be a drug effect targeting the underlying disease mechanisms. We strongly advise patients and physicians not to use cyclophosphamide for ME/CFS patients outside of clinical trials before a randomized trial has been conducted, to evaluate the possible benefits of the drug.

## Conclusion

This study shows that cyclophosphamide intervention is feasible for ME/CFS patients. The growing evidence for immune alterations in ME/CFS and the high symptom burden with very low quality of life, we believe can justify use of an immune modulating drug with possible side effects. The treatment period was demanding for most patients, but in total the toxicity was interpreted as acceptable. The treatment was associated with long-lasting improvements of ME/CFS symptoms for approximately half of patients. However, due to the lack of a placebo group, response data must be interpreted with great caution. In the further work to find effective treatment, we will consider a new multicenter, randomized, double-blind and placebo-controlled trial with cyclophosphamide. Should this trial prove cyclophosphamide to be beneficial for ME/CFS patients, this could also be important in the search for relevant disease mechanisms.

## Data Availability Statement

The datasets generated from this study are available on reasonable request to the corresponding author.

## Ethics Statement

The studies involving human participants were reviewed and approved by The Regional Committees for Medical and Health Research Ethics (2014/1672), and by the National Medicines Agency in Norway. The patients/participants provided their written informed consent to participate in this study.

## Author Contributions

IR, ØF, KS, and OM: conception and design. IR, ØF, AF, KT, AL, MV, BL, and OM: analyses and interpretation. IR, ØF, AF, IK-V, MH, KS, and OM: inclusion and follow-up of patients. IR, ØF, AF, IK-V, KS, KR, KA, MH, OM, AL, MV, and BL: collection and assembly of data. IR, ØF, KS, KR, KA, OD, and OM: administrative, technical, biobank and logistic support. IR, ØF, AF, KS, and OM: drafting the article. All authors: critical revision of the article and final approval of the manuscript.

## Conflict of Interest

The authors declare that the research was conducted in the absence of any commercial or financial relationships that could be construed as a potential conflict of interest.

## Supplementary Material

Supplementary Figure 1 SF-36 subscales (raw scores) and Fatigue Severity Scale during follow-up until 18 months, shown for the Intention-to-treat population (*n* = 40) in **(A,C,E,G)**, and for responders (*n* = 22) vs. non-responders (*n* = 18) in **(B,D,F,H)**. The SF-36 subscales for Vitality **(A,B)**, Social Function **(C,D)**, Bodily Pain **(E,F)**, and Fatigue Severity Scale **(G,H)** are shown. SF-36 subscales with scale 0–100, higher number indicates better function. Fatigue Severity Scale with scores 7–63, higher score indicates more fatigue. SF-36, Short Form 36; CI, confidence intervals; SD, standard deviation.

Supplementary Table 1 Medical history and concomitant diseases reported at baseline, shown by System Organ Class (SOC) and CTCAE term.

Supplementary Table 2 Previous treatments for ME/CFS, reported at baseline.

Supplementary Table 3 Concomitant medication during 18 months follow-up (shown by ATC-code).

Supplementary Table 4 Serious Adverse Events during 18 months follow-up (System Organ Class, CTCAE term, SAE category and relation to treatment). **Trial protocol**.

Data Sheet 1 Trial protocol.
