# Supplementary figures and images for: Intravenous Cyclophosphamide in Myalgic Encephalomyelitis/Chronic Fatigue Syndrome. An Open-Label Phase II Study
Source: Front Med (Lausanne). 2020 Apr 29;7:162. doi: 10.3389/fmed.2020.00162 (PMC7201056; doi:10.3389/fmed.2020.00162)

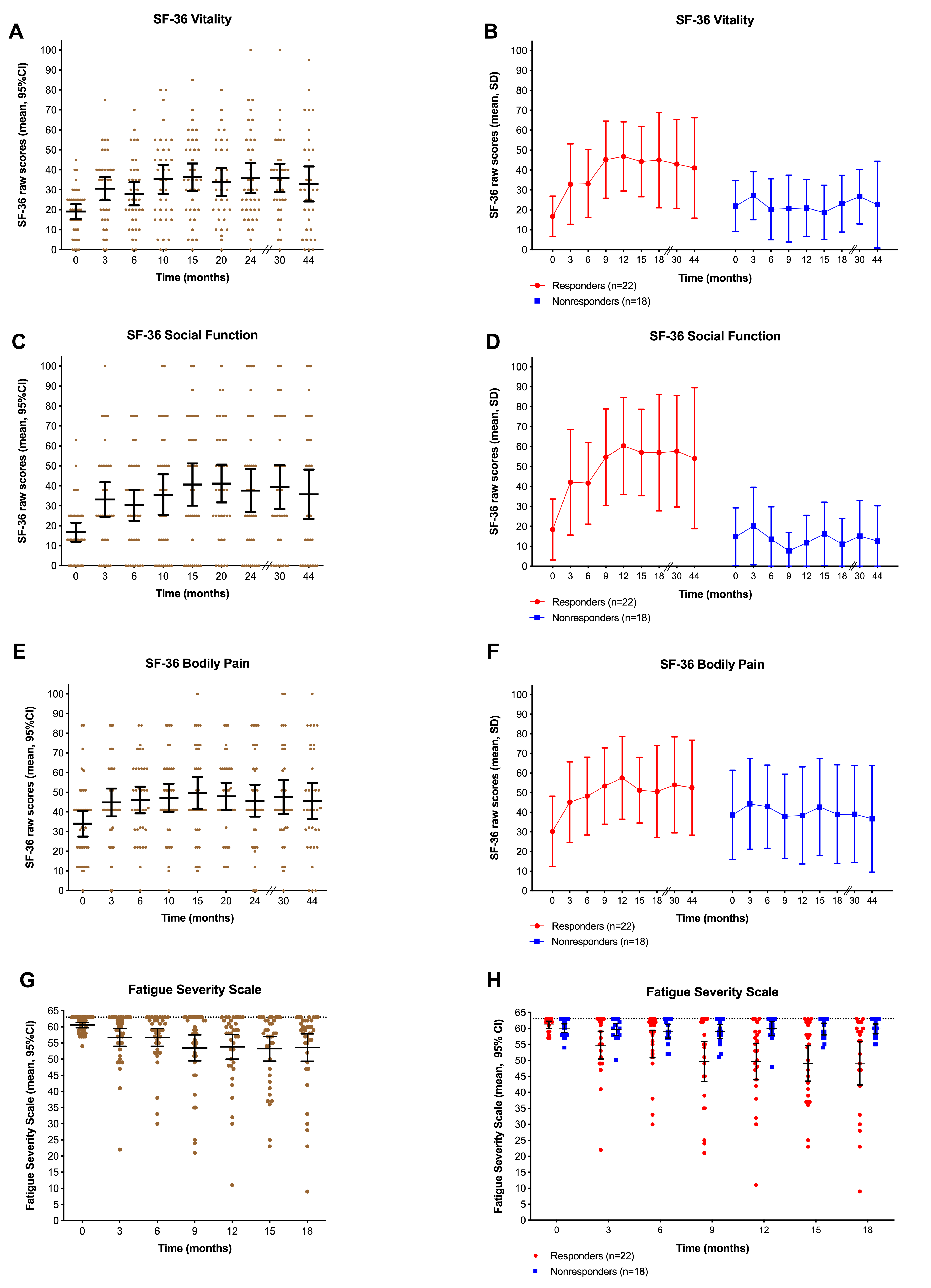

Supplement: Supplementary Figure 1 — SF-36 subscales (raw scores) and Fatigue Severity Scale during follow-up until 18 months, shown for the Intention-to-treat population (n = 40) in (A,C,E,G), and for responders (n = 22) vs. non-responders (n = 18) in (B,D,F,H). The SF-36 subscales for Vitality (A,B), Social Function (C,D), Bodily Pain (E,F), and Fatigue Severity Scale (G,H) are shown. SF-36 subscales with scale 0–100, higher number indicates better function. Fatigue Severity Scale with scores 7–63, higher score indicates more fatigue. SF-36, Short Form 36; CI, confidence intervals; SD, standard deviation. [file Image_1.TIFF]
