# Supplementary material for: Intravenous Cyclophosphamide in Myalgic Encephalomyelitis/Chronic Fatigue Syndrome. An Open-Label Phase II Study
Source: Front Med (Lausanne). 2020 Apr 29;7:162. doi: 10.3389/fmed.2020.00162 (PMC7201056; doi:10.3389/fmed.2020.00162)
Supplement: Supplementary Table 1 — Medical history and concomitant diseases reported at baseline, shown by System Organ Class (SOC) and CTCAE term. [file Table_1.DOCX]

*Supplementary Table 1.* Medical history and concomitant diseases reported at baseline, shown by System Organ Class (SOC) and CTCAE term

| *System Organ Class (SOC)* | *CTCAE term* | *n* | *%* |
| --- | --- | --- | --- |
| Endocrine disorders | Hypothyroidism | 4 | 10.0 |
| Immune system disorder | Allergy | 16 | 40.0 |
| Musculoskeletal and connective tissue disorder | Fibromyalgia | 3 | 7.5 |
| Psychiatric disorder | Depression | 4 | 10.0 |
| Psychiatric disorder | Anxiety | 4 | 10.0 |
| Other | Other | 14 | 35.0 |
