# Supplementary material for: Intravenous Cyclophosphamide in Myalgic Encephalomyelitis/Chronic Fatigue Syndrome. An Open-Label Phase II Study
Source: Front Med (Lausanne). 2020 Apr 29;7:162. doi: 10.3389/fmed.2020.00162 (PMC7201056; doi:10.3389/fmed.2020.00162)
Supplement: Supplementary Table 2 — Previous treatments for ME/CFS, reported at baseline. [file Table_2.docx]

*Supplementary Table 2.* Previous treatments for ME/CFS, reported at baseline.

| *Type of treatment, n (%)* | *n* | *%* |  |
| --- | --- | --- | --- |
| Cognitive therapy (CT) |  |  |  |
| “Lightning Process” (LP) | 13 | 32.5 |  |
| Mindfulness | 11 | 27.5 |  |
| Other CT | 4 | 10.0 |  |
| Any CT (LP, Mindfulness, Other) | 21 | 52.5 |  |
| Physical therapy |  |  |  |
| Graded exercise therapy (GET) | 6 | 15.0 |  |
| Other physical therapy | 15 | 37.5 |  |
| GET or other physical therapy | 18 | 45.0 |  |
| Activity management (adaptive pacing) | 15 | 37.5 |  |
| Not received any of these treatments | 8 | 20.0 |  |
| Not answered | 1 | 2.5 |  |
| Medical treatments |  |  |  |
| Nexavir | 2 | 5.0 |  |
| Vitamin B12-injections | 16 | 40.0 |  |
| Long term antibiotics | 10 | 25.0 |  |
| Low dose naltrexone | 15 | 37.5 |  |
| Rituximab | 15 | 37.5 |  |
| Not received any of these treatments | 7 | 17.5 |  |
| Not answered | 1 | 2.5 |  |
