# Supplementary material for: Intravenous Cyclophosphamide in Myalgic Encephalomyelitis/Chronic Fatigue Syndrome. An Open-Label Phase II Study
Source: Front Med (Lausanne). 2020 Apr 29;7:162. doi: 10.3389/fmed.2020.00162 (PMC7201056; doi:10.3389/fmed.2020.00162)
Supplement: Supplementary Table 3 — Concomitant medication during 18 months follow-up (shown by ATC-code). [file Table_3.DOCX]

*Supplementary Table 3.* Concomitant medication during 18 months follow-up (shown by ATC-code)

| *ATC code* | *Description* | *N=40* |  |
| --- | --- | --- | --- |
| A02 | Antacids, n (%) | 9 (22.5) |  |
| A03, A04 | Antiemetics, n (%) | 10 (25.0) |  |
| A06 | Laxantia, n (%) | 2 (5.0) |  |
| B01 | Antithrombotic agents, n (%) | 1 (2.5) |  |
| B03A, B03BB | Vitamin B12 supplements, n (%) | 5 (12.5) |  |
| C07 | Betablockers, n (%) | 3 (7.5) |  |
| C08, C09 | Antihypertensive agents, n (%) | 4 (10.0) |  |
| C10 | Statins, n (%) | 2 (5.0) |  |
| G01, J01-05 | Antibiotics, n (%) | 13 (32.5) |  |
| G03A | Contraceptives (systemic), n (%) | 4 (10.0) |  |
| H03 | Thyroid hormone replacement, n (%) | 4 (10.0) |  |
| M01A | NSAID, n (%) | 17 (42.5) |  |
| N02A | Opioids^a^, n (%) | 11 (27.5) |  |
| N02B | Paracetamol, n (%) | 15 (37.5) |  |
| N02C | Antimigraine agents, n (%) | 5 (12.5) |  |
| N03A | Antiepileptic agents, n (%) | 1 (2.5) |  |
| N05B | Anxiolytica, n (%) | 4 (10.0) |  |
| N05C | Hypnotics and sedatives^b^, n (%) | 20 (50.0) |  |
| N06 | Antidepressants, n (%) | 6 (15.0) |  |
| R01, R03, R06A, S01G | Allergy and asthma medications, n (%) | 20 (50.0) |  |
|  | Other medications, n (%) | 30 (75.0) |  |
|  | Dietary supplements (non-ATC) , n (%) | 10 (25.0) |  |

^a^: 11 out of 40 patients received opioids at any time during follow-up. Among these, only 2 used tramadol daily on a regular basis, and 9 used codeine phosphate or tramadol on demand. None of the patients used any stronger opioids. ^b^: 20 out of 40 patients had used hypnotics regularly or sporadically at any time during follow-up, 4 of whom had tried more than one type of hypnotic. Among the 20, 10 had used melatonin, 10 had used zopiclone and 4 patients had used nitrazepam or zolpidem.
