# Supplementary material for: Intravenous Cyclophosphamide in Myalgic Encephalomyelitis/Chronic Fatigue Syndrome. An Open-Label Phase II Study
Source: Front Med (Lausanne). 2020 Apr 29;7:162. doi: 10.3389/fmed.2020.00162 (PMC7201056; doi:10.3389/fmed.2020.00162)
Supplement: Supplementary Table 4 — Serious Adverse Events during 18 months follow-up (System Organ Class, CTCAE term, SAE category and relation to treatment). Trial protocol. [file Table_4.DOCX]

*Supplementary Table 4.* Serious Adverse Events during 18 months follow-up (System Organ Class, CTCAE term, SAE category and relation to treatment)

| *System Organ Class* | *CTCAE term* | *Grade* | *Adverse event category* | *Relation to treatment* |
| --- | --- | --- | --- | --- |
| Cardiac disorders | Sinus tachycardia | 3 | SUSAR/ Hospitalization | Probable |
|  | Sinus tachycardia | 3 | SAE/ Hospitalization | Unlikely |
| Gastrointestinal disorders | Stomach pain | 2 | SAE/ Hospitalization | Possible |
| General disorders | Other (ME/CFS symptom exacerbation) | 3 | SAE/ Hospitalization | Probable |
| Infections and infestations | Urinary tract infection | 3 | SAE/ Hospitalization | Possible |
|  | Sepsis† | 4 | SAE/ Hospitalization | No |
|  | Sepsis† | 4 | SAE/ Hospitalization | No |
|  | Upper respiratory infection | 3 | SAE/ Hospitalization | No |
| Metabolism and nutrition disorders | Dehydration | 3 | SAE/ Hospitalization | Probable |
| Neoplasms | Other (olfactory meningioma) * | 3 | SAE/ Hospitalization | No |
| Renal and urinary disorders | Renal calculi† | 3 | SAE/ Hospitalization | No |
| Skin and subcutaneous tissue disorders | Urticaria | 3 | SAE/ Hospitalization | Possible |

† 3 hospitalizations for one study patient; complications after elective daytime surgery.

* Elective hospitalizations/procedures
